# Writing an effective literature review: Part I: Mapping the gap

**DOI:** 10.1007/s40037-017-0401-x

**Published:** 2017-12-19

**Authors:** Lorelei Lingard

**Affiliations:** Schulich School of Medicine & Dentistry, Health Sciences Addition, Western University, London, Ontario Canada

In the Writer’s Craft section we offer simple tips to improve your writing in one of three areas: Energy, Clarity and Persuasiveness. Each entry focuses on a key writing feature or strategy, illustrates how it commonly goes wrong, teaches the grammatical underpinnings necessary to understand it and offers suggestions to wield it effectively. We encourage readers to share comments on or suggestions for this section on Twitter, using the hashtag: #how’syourwriting?

This Writer’s Craft instalment is the first in a two-part series that offers strategies for effectively presenting the literature review section of a research manuscript. This piece alerts writers to the importance of not only summarizing what is known but also identifying precisely what is not, in order to explicitly signal the relevance of their research. In this instalment, I will introduce readers to the mapping the gap metaphor, the knowledge claims heuristic, and the need to characterize the gap.

## Mapping the gap

The purpose of the literature review section of a manuscript is not to report what is known about your topic. The purpose is to identify what remains *unknown—*what academic writing scholar Janet Giltrow has called the ‘knowledge deficit’*—*thus establishing the need for your research study [1]. In an earlier Writer’s Craft instalment, the Problem-Gap-Hook heuristic was introduced as a way of opening your paper with a clear statement of the problem that your work grapples with, the gap in our current knowledge about that problem, and the reason the gap matters [2]. This article explains how to use the literature review section of your paper to build and characterize the Gap claim in your Problem-Gap-Hook. The metaphor of ‘mapping the gap’ is a way of thinking about how to select and arrange your review of the existing literature so that readers can recognize why your research needed to be done, and why its results constitute a meaningful advance on what was already known about the topic.

Many writers have learned that the literature review should describe what is known. The trouble with this approach is that it can produce a laundry list of facts-in-the-world that does not persuade the reader that the current study is a necessary next step. Instead, think of your literature review as painting in a map of your research domain: as you review existing knowledge, you are painting in sections of the map, but your goal is not to end with the whole map fully painted. That would mean there is nothing more we need to know about the topic, and that leaves no room for your research. What you want to end up with is a map in which painted sections surround and emphasize a white space, a gap in what is known that matters. Conceptualizing your literature review this way helps to ensure that it achieves its dual goal: of presenting what is known and pointing out what is not—the latter of these goals is necessary for your literature review to establish the necessity and importance of the research you are about to describe in the methods section which will immediately follow the literature review.

To a novice researcher or graduate student, this may seem counterintuitive. Hopefully you have invested significant time in reading the existing literature, and you are understandably keen to demonstrate that you’ve read everything ever published about your topic! Be careful, though, not to use the literature review section to regurgitate all of your reading in manuscript form. For one thing, it creates a laundry list of facts that makes for horrible reading. But there are three other reasons for avoiding this approach. First, you don’t have the space. In published medical education research papers, the literature review is quite short, ranging from a few paragraphs to a few pages, so you can’t summarize everything you’ve read. Second, you’re preaching to the converted. If you approach your paper as a contribution to an ongoing scholarly conversation,[2] then your literature review should summarize just the aspects of that conversation that are required to situate *your* conversational turn as informed and relevant. Third, the key to relevance is to point to *a gap* in what is known. To do so, you summarize what is known for the express purpose of *identifying what is not known*. Seen this way, the literature review should exert a gravitational pull on the reader, leading them inexorably to the white space on the map of knowledge you’ve painted for them. That white space is the space that your research fills.

## Knowledge claims

To help writers move beyond the laundry list, the notion of ‘knowledge claims’ can be useful. A knowledge claim is a way of presenting the growing understanding of the community of researchers who have been exploring your topic. These are not disembodied facts, but rather incremental insights that some in the field may agree with and some may not, depending on their different methodological and disciplinary approaches to the topic. Treating the literature review as a story of the knowledge claims being made by researchers in the field can help writers with one of the most sophisticated aspects of a literature review—locating the knowledge being reviewed. Where does it come from? What is debated? How do different methodologies influence the knowledge being accumulated? And so on.

Consider this example of the knowledge claims (KC), Gap and Hook for the literature review section of a research paper on distributed healthcare teamwork:

*KC: We know that poor team communication can cause errors.*

*KC: And we know that team training can be effective in improving team communication.*

*KC: This knowledge has prompted a push to incorporate teamwork training principles into health professions education curricula.*

*KC: However, most of what we know about team training research has come from research with co-located teams—i. e., teams whose members work together in time and space.*

*Gap: Little is known about how teamwork training principles would apply in distributed teams, whose members work asynchronously and are spread across different locations.*

*Hook: Given that much healthcare teamwork is distributed rather than co-located, our curricula will be severely lacking until we create refined teamwork training principles that reflect distributed as well as co-located work contexts.*

The ‘We know that …’ structure illustrated in this example is a template for helping you draft and organize. In your final version, your knowledge claims will be expressed with more sophistication. For instance, ‘We know that poor team communication can cause errors’ will become something like ‘Over a decade of patient safety research has demonstrated that poor team communication is the dominant cause of medical errors.’ This simple template of knowledge claims, though, provides an outline for the paragraphs in your literature review, each of which will provide detailed evidence to illustrate a knowledge claim. Using this approach, the order of the paragraphs in the literature review is strategic and persuasive, leading the reader to the gap claim that positions the relevance of the current study. To expand your vocabulary for creating such knowledge claims, linking them logically and positioning yourself amid them, I highly recommend Graff and Birkenstein’s little handbook of ‘templates’ [3].

As you organize your knowledge claims, you will also want to consider whether you are trying to map the gap in a well-studied field, or a relatively understudied one. The rhetorical challenge is different in each case. In a well-studied field, like professionalism in medical education, you must make a strong, explicit case for the existence of a gap. Readers may come to your paper tired of hearing about this topic and tempted to think we can’t possibly need more knowledge about it. Listing the knowledge claims can help you organize them most effectively and determine which pieces of knowledge may be unnecessary to map the white space your research attempts to fill. This does not mean that you leave out relevant information: your literature review must still be accurate. But, since you will not be able to include everything, selecting carefully among the possible knowledge claims is essential to producing a coherent, well-argued literature review.

## Characterizing the gap

Once you’ve identified the gap, your literature review must characterize it. What *kind* of gap have you found? There are many ways to characterize a gap, but some of the more common include:

- a pure knowledge deficit—‘no one has looked at the relationship between longitudinal integrated clerkships and medical student abuse’
- a shortcoming in the scholarship, often due to philosophical or methodological tendencies and oversights—‘scholars have interpreted x from a cognitivist perspective, but ignored the humanist perspective’ or ‘to date, we have surveyed the frequency of medical errors committed by residents, but we have not explored their subjective experience of such errors’
- a controversy—‘scholars disagree on the definition of professionalism in medicine …’
- a pervasive and unproven assumption—‘the theme of technological heroism—technology will solve what ails teamwork—is ubiquitous in the literature, but what is that belief based on?’

To characterize the kind of gap, you need to know the literature thoroughly. That means more than understanding each paper individually; you also need to be placing each paper in relation to others. This may require changing your note-taking technique while you’re reading; take notes on what each paper contributes to knowledge, but also on how it relates to other papers you’ve read, and what it suggests about the kind of gap that is emerging.

In summary, think of your literature review as mapping the gap rather than simply summarizing the known. And pay attention to characterizing the kind of gap you’ve mapped. This strategy can help to make your literature review into a compelling argument rather than a list of facts. It can remind you of the danger of describing so fully what is known that the reader is left with the sense that there is no pressing need to know more. And it can help you to establish a coherence between the kind of gap you’ve identified and the study methodology you will use to fill it.
